# Expression of a Secreted Fibroblast Growth Factor Binding Protein-1 (FGFBP1) in Angioproliferative Kaposi Sarcoma

**DOI:** 10.4172/2155-6113.1000309

**Published:** 2014-06

**Authors:** Patricio E Ray, Ali Al-Attar, Xue-Hui Liu, Jharna R Das, Elena Tassi, Anton Wellstein

**Affiliations:** 1 Children’s Research Institute, Children’s National Medical Center, The George Washington University, Washington DC, USA; 2 Lombardi Cancer Center, Georgetown University, Washington DC, USA

**Keywords:** Fibroblast growth factor, HIV-Tat, Kaposi’s sarcoma, Endothelial cells, KS spindle cells, FGF-2 release, Pediatric AIDS, FGF- 2 binding, Angiogenesis

## Abstract

**Objective:**

Kaposi’s sarcoma (KS) is an angioproliferative disease frequently seen in patients with the acquired immunodeficiency syndrome (AIDS). Previous studies suggest that the HIV-1 protein Tat and Fibroblast Growth Factor 2 (FGF-2) have synergistic angiogenic effects in AIDS-KS tumors. However, the mechanisms by which FGF-2 is released and activated in KS tumors are not clearly defined. We carried out this study to determine whether an FGF-binding protein (FGFBP1 or BP1) that enhances the angiogenic activity of FGF-2 is expressed in AIDS-KS tumors, and to define whether BP1, FGF-2, and HIV-Tat protein-protein interactions could play a potential clinically role in the pathogenesis of AIDS-KS.

**Methods:**

BP1 was localized in AIDS-KS lesions by immunohistochemistry and in situ hybridization studies. The binding of radiolabeled FGF-2 to His-tagged BP1 or the FGF-receptor 1 was assessed in the presence and absence of HIV-Tat and other viral proteins. Mice carrying tetracycline-regulated BP1 transgene mice were used to determine whether activation of BP1 during wound healing induces KS-like lesions.

**Results:**

BP1 expression was detected in AIDS-KS tumor keratinocytes, spindle cells, and infiltrating mononuclear cells. In addition, HIV-Tat competed for the binding of FGF-2 to immobilized BP1, but does not affect the interactions of FGF-2 with its high affinity receptor (FGFR-1). In contrast, two other HIV-proteins, Nef and gp120, did not affect the binding of FGF-2 to BP1 or to FGFR-1. Finally, up-regulation of BP1 expression in tetracycline-regulated –conditional BP1 transgenic mice subjected to skin wounds, induced KS-like skin lesions.

**Conclusion:**

Taking into consideration the results of previous studies showing that both HIV-Tat and BP1 enhance the mitogenic and angiogenic activity of locally-stored FGF-2, both *in vitro* and *in vivo*, our findings suggest a novel mechanism by which the release and activity of FGFs can be modulated in AIDS-KS tumors by HIV-Tat as well as BP1.

## Introduction

Kaposi’s Sarcoma (KS) is an angiogenic multifocal proliferative tumor which can arise in the skin, mucosa surfaces, and internal organs [1]. Elderly men of the Eastern- Mediterranean origin, Blacks living in the Sub-Saharan region, immunosupressed patients, and homosexual or bisexual men infected with the human immunodeficiency virus type 1, are at high risk of developing different clinical and epidemiological forms of KS (classical KS, African KS, post-transplant KS, and AIDS-KS respectively) [1]. Infections with the Kaposi’s Sarcoma Associated Herpes Virus (KSHV or HHV-8) play a key role in the development and pathogenesis of KS by inducing the proliferation of cells and release of angiogenic growth factors [2,3]. Histologically, KS lesions are characterized by proliferating spindle cells, angiogenesis, erythrocyte-replete vascular slits, profuse edema, and a variable inflammatory cell infiltrate [1]. KS spindle cells and infiltrating inflammatory cells elaborate a variety of pro-inflammatory and angiogenic factors which are thought to induce the progression of KS by autocrine and paracrine mechanisms [4].

Among the many cytokines accumulated in KS lesions, Fibroblast Growth Factor 2 (FGF-2 or basic FGF), plays an important role in the pathogenesis of KS [5,6]. FGF-2 is a potent angiogenic growth factor which lacks a classic signal peptide for secretion and is released from cells by non-conventional pathways [7–9]. FGF-2 also synergizes with the HIV-1 protein Tat to promote angiogenesis and tumor growth in AIDS-KS lesions [5] and Tat has been shown to be able to induce angiogenesis. Interestingly, the release of biologically active FGF-2 is controlled by neighboring as well as invading inflammatory cells. A major control mechanism of FGF-2 local paracrine activity is extracellular sequestration followed by its selective release [8,9]. Heparan sulfate proteoglycans (HSPG) in the extracellular matrix are used to immobilize and store FGF-2 and other heparin binding proteins [8–11] including HIV-Tat [12,13]. Tat may also exit HIV-infected cells via non-conventional pathways, and binds to HSPG with an affinity similar to FGF-2 [13]. Many biological activities of Tat in cultured cells, such as induction of endothelial cell growth, migration, and invasion, are enhanced by low concentrations and inhibited by high concentrations of heparin, as has been shown for FGF-2 [12,13]. However, the mechanisms by which FGF-2 or HIV-Tat are released and/or activated in KS lesions is not fully understood. In previous studies we have found that a Fibroblast Growth Factor Binding Protein (FGFBP1 or BP1), which was first identified in a tumor cell line [14], is a carrier molecule for FGF-2. Subsequent studies demonstrated that BP1 binding releases FGF-2 from the HSPG and can transport bound FGF-2 to the target cell surface [15,16]. Taken together, these findings suggest that FGF-2, BP1, and HIV-Tat may have significant overlapping biological interactions in the pathogenesis of KS. Thus, we carried out this study to determine whether BP1 is expressed in KS lesions, and to define whether BP1, FGF-2, and HIV-Tat have significant protein-protein interactions that could play a potential clinically role in the pathogenesis of AIDS-KS.

## Methods

### Patients

This study was approved by the Institutional Review Board from the Children’s National Medical Center. The diagnosis of HIV-1 infection and KS were done by the attending physicians following standard clinical and pathologic criteria. KS tissues were obtained from biopsies or autopsies of patients with AIDS-KS lesions in the skin (n =2), lung (n=1), gastrointestinal tract (n=1), and from one KS tumor harvested from the skin of an HIV-negative patients. Control sections were obtained from AIDS patients without KS: skin (n =2); liver (n=1), lung (n=1), gastrointestinal tract (n=1).

### Immunohistochemisty

Tissues were fixed in 10% neutral buffered formalin and embedded in paraffin. Paraffin sections were cut at 4 μm, deparaffinized and rehydrated. Sections were then heated twice for 5 min each in 0.01 M sodium citrate (pH 6.0) in a microwave oven (2450 mHz, 850 w) to augment antigen retrieval. Endogenous peroxidase activity was blocked by treating with 3% H2O2 in 100% methanol for 10 min. Immunostaining was performed with a commercial streptavidin-biotin-peroxidase complex HistostainTM SP kit (Zymed, South San Francisco, CA, USA) according to the manufacture’s instructions as previously described [17]. We used a rabbit polyclonal BP1 antibody diluted 1:50 in 2% BSA PBS [17]. The specificity of this BP1 antibody has been demonstrated before [17,18]. To determine the localization of FGF-2 we used a monoclonal antibody against bovine FGF-2 from Upstate Biotechnology (Lake Placid, NY, USA). Different cell types in the KS lesions were identified with antibodies against von Willebrand factor (vWF) and CD68, for mononuclear cells, all from Dako (Carpinteria, CA, USA). The co-localization of BP1 in infiltrating mononuclear cells was performed by using Histostain-DSTM double staining kit (Zymed). Controls sections included replacing the primary antibody with equivalent concentrations of mouse non immune IgG, or a control polyclonal rabbit immunoglobin preparation derived from non-immune rabbit, and omitting the first antibodies.

### In situ hybridization

In situ hybridization studies for FGF-BP were done in 4 μm sections as previously described [17]. Briefly, these sections were heat-fixed for 30 min. at 65°C and deparaffinized. After a 30-min. incubation in 5 mmol/L levamisole, they were washed in PBS and in DEPC-treated water for 5 min., immersed in 0.2 mol/L HCl for 20 min, and digested with 20–40 μg/ml proteinase K (Sigma) for 10 min. at 37°C. Slides were hybridized with a 790-bp human FGF-BP RNA probe (Gene Bank # M60047). This fragment was subcloned into the Hind III and XbaI sites of the pRC/ CMV vector between T7 and SP6 promoters as previously described [17]. For both probes, *in vitro* transcription reactions were performed with SP6 or T7 RNA polymerase in the presence of DIG-UTP by using a DIG RNA labeling Kit (Boehringer-Mannheim Biochemica). Labeling efficiency of the riboprobe was estimated by comparison with 10-fold serial dilution of a digoxigenin-labeled control riboprobe and direct detection of the labeled riboprobe with antidigoxigenin antibodies. Riboprobe concentrations were adjusted to be equivalent on the basis of the labeling efficiency before use in the in situ hybridization studies. The BP1 probe was used at a final concentration of 0.1–0.5 ng/μl in hybridization buffer. Subsequently the slides were washed and incubated with anti-digoxigenin antibody conjugated with alkaline phosphatase (Boehringer- Mannheim Biochemica) at the dilution of 1:750 for 30 min. at 37°C. The negative controls included: (1) hybridization with the sense probe, (2) RNase A (100 μg/ml in 10 mM Tris HCl, pH 8.0,1 mM EDTA) pretreatment before hybridization, and (3) omission of either the antisense RNA probe or the anti-digoxigenin antibody.

### Binding studies

Briefly, 96 wells plates (EIA/RIA Strip Plate, Corning Inc. Corning NY) were coated with Histidine-tagged BP1 (0.1 ng/ml) diluted in Tris Buffered Saline. Excess of unbound His-BP was removed by washing. Non-specific binding was blocked by adding 300 μl (LB media) and further washes. [125]I-FGF-2 (1–20 μg/ml) was added to the wells at RT0 with constant rocking for two hours, and then, unbound FGF-2 was removed by washing. The binding of radiolabeled FGF-2 to His-BP1 was measured by counting radioactive emission from the individual wells. In some experiments we added HIV-Tat (1–3000 ng/ml) together with (1–20 μg/ml) FGF-2, to determine whether Tat can compete with FGF-2 for its binding to BP-FGF, or 1 hr later after addition of [125] I-FGF-2, to determine whether Tat can dissociate the complex FGF-2/BP1. To determine the specificity of Tat activity other experiments were done in the presence or absence of Nef and gp120 (1–3000 ng/ml). The HIV-1 recombinant peptides were provided by National Institute of Allergy and Infectious, Diseases, NIH AIDS Research and Reference Reagent Program: Tat (contributor Dr. John Brady); SIVnef (contributor Dr. Jose Torres); HIV-1IIIB gp120 (contributor NIAID, produced by ImmunoDiagnostics, Inc). [125]-I-FGF2 was purchased from Amersham Pharmacia Biotech (Piscataway, NJ). Human recombinant FGF-2 was purchased from Life Technologies, Inc (Gaithersburg, MD). Recombinant histidine-tagged BP1 protein purification was produced in Sf-9 insect cells with a baculovirus vector that contains an expression cassette for human BP1 (BAC-To-BAC Baculovirus Expression System, Life Technologies Inc., Gaithersburg, MD) as previously described (15–16). In addition, binding studies of [125]I-FGF-2 to its high and low affinity receptors were done in the presence or absence of BP1. These studies utilized recombinant FGFR-1/Fc chimera (from R &D Systems Inc., Catalog # 658-FR, Minneapolis, MN), containing the extracellular domain of human FGFR-1 fused via a polypeptide linker to the carboxy-terminal Fc region of human IgG1. The binding interactions between BP1 and HIV-Tat were evaluated by using saturation binding curves and by competitive displacement of the [125I] FGF–2 ligand with an excess of cold ligand (Tat or BP1).

### BP1 transgenic mice

The generation of the tetracycline-regulated conditional BP1 transgenic mice, and the impact of BP1 on angiogenesis and wound healing, were previously described by Tassi et al. [19]. BP1 transgene (Tg) expression is silenced by feeding these mice a diet supplemented with an orally available tetracycline (e.g. doxycycline) from Bio-Serv (Frenchtown NH) (BP1 OFF) and induced by a switch to regular mouse chow that lacks tetracycline (BP1 ON), typically for 2 weeks before the experiments. The dorsal skin of BP1 OFF and BP1 ON animals was injured by a full-thickness dermal biopsy punch (3 mm diameter) under anesthesia, as previously described [19]. Histological sections were then screened for the presence / absence of KS-like lesions. Immunohistochemistry studies were done to detect the infiltration of macrophages using the F4/80 antibody (Serotec, Raleigh, NC) as described by Tassi et al. [19]. Animals experiments were reviewed and approved by the Institutional Animal Care and Use Committee at both institutions, Children’s National Medical Center, and Lombardi Cancer Center, at Georgetown University, Washington DC.

### Statistical analyses

Results are expressed as the mean value + SD. Difference between two groups were compared by Students’t test. P-values less than 0.05 were considered significant. When more than two means were compared, difference was measured by one way analysis of variance followed by multiple comparisons using the Student-Neuman-Keul’s test.

## Results

### BP1 expression in AIDS Kaposi’s sarcoma (KS)

**Figure 1:**
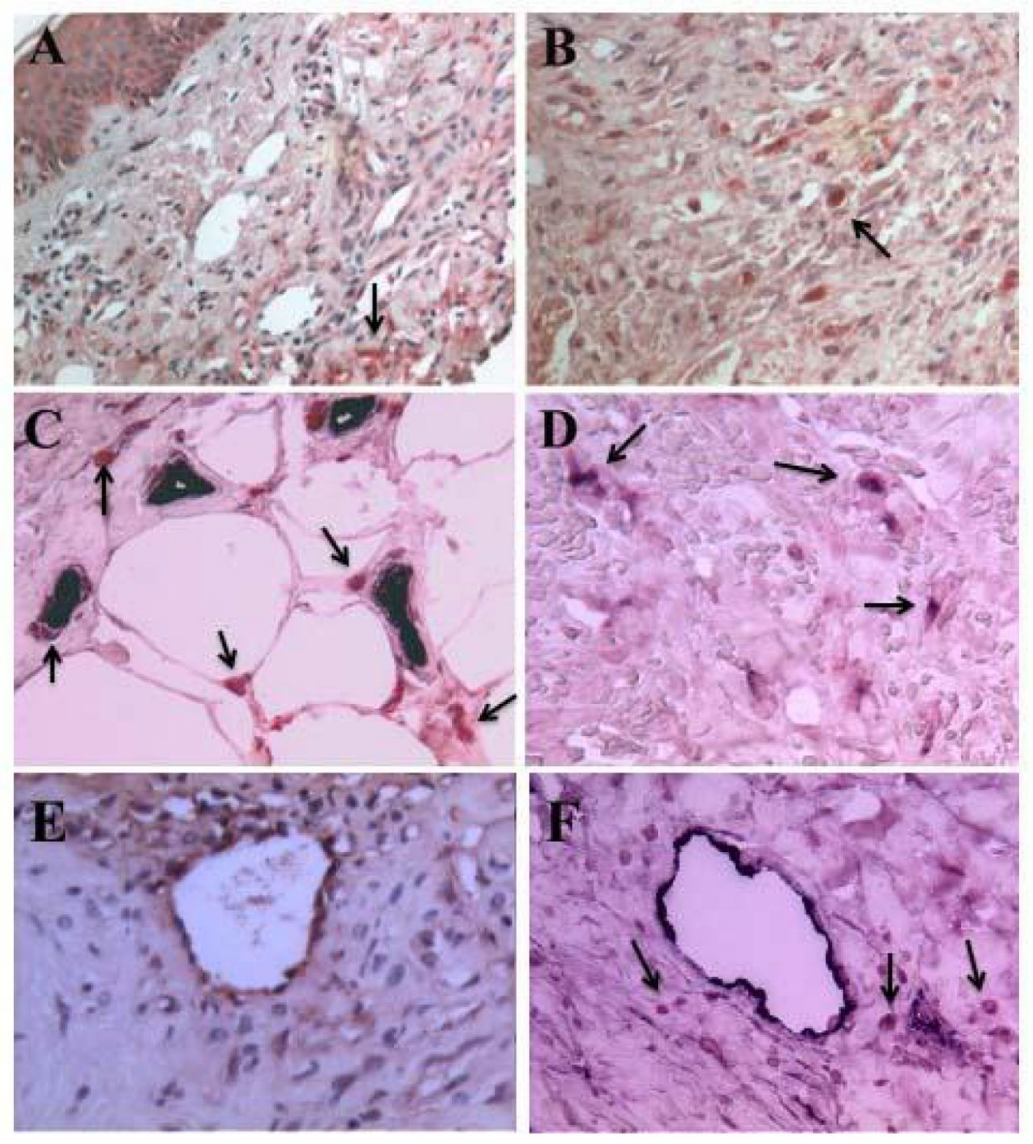
Detection of BP1 and FGF-2 in Kaposi’s sarcoma skin lesions by immunohistochemistry Panels A and B show localization of BP1 protein (red color) in AIDS-KS skin lesions pointed by the black arrows (A, 200X; B, 500X). Panel C shows capillary endothelial cells stained positive with vWF (dark color) located in close proximity to infiltrating cells expressing BP1 (red color) and pointed by the black arrows (500X). Panel D shows co-localization of BP1 (red color) in infiltrating mononuclear cells stained also with an antibody against the CD-68 antigen (dark color) in a KS skin lesions (500X). Dual BP1 and CD68 positive cells are pointed by the black arrows. Panel E, shows the localization of FGF-2 (red color) in an AIDS-KS skin lesion. FGF-2 was localized in endothelial cells, infiltrating spindle cells, and the extracellular matrix (500X). Panel F shows vWF positive endothelial cells in an AIDS-KS skin tumor (dark color), and infiltrating BP1 positive cells (red color) surrounding the vessels. Black arrows point to the BP1 positive cells (500X).

**Figure 2:**
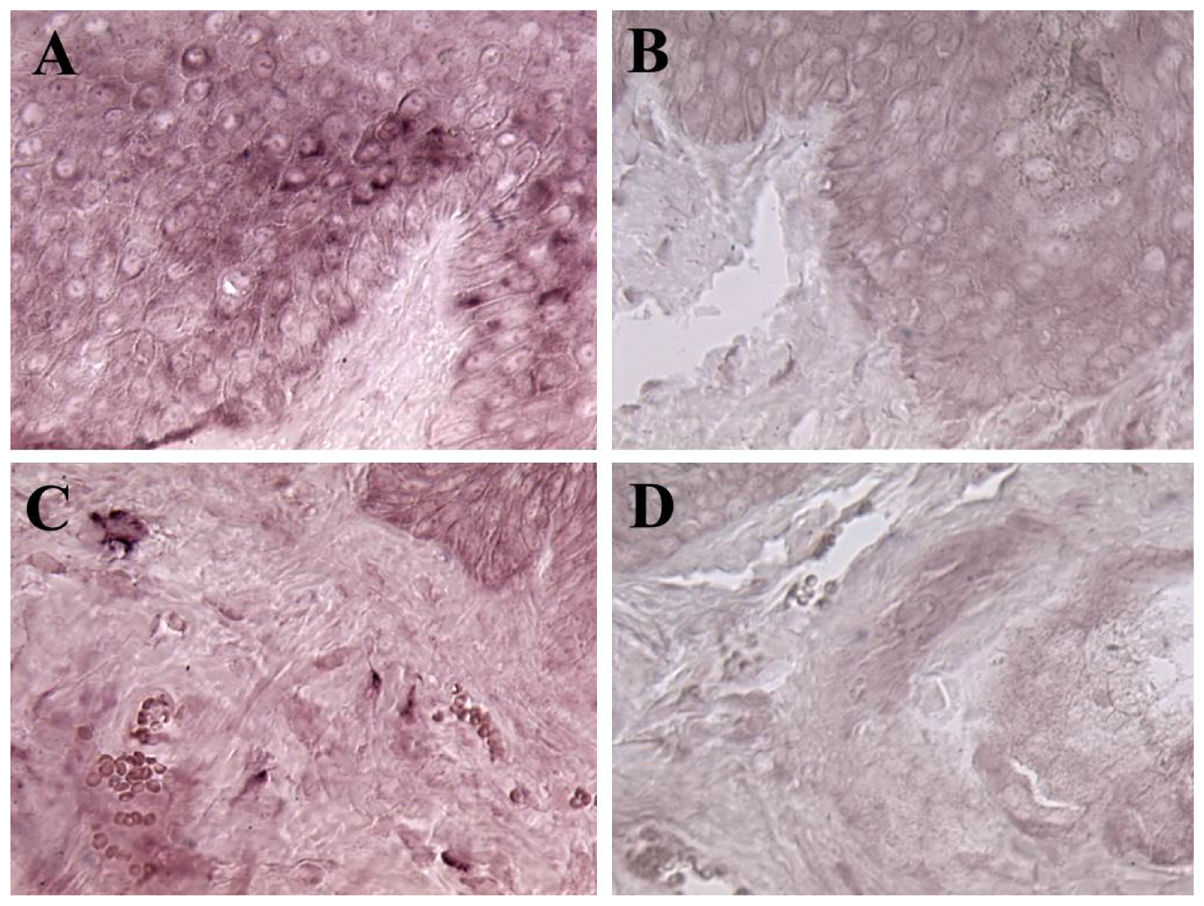
Detection of BP1 mRNA in a Kaposi’s sarcoma skin lesions by in situ hybridization Panels A and C show localization of BP1 mRNA (dark violet color) in representative KS sections hybridized with the antisense BP1 probe. Panels B and D show similar KS sections hybridized with the sense BP1 probe (Original magnification 500X).

Since FGF-2 was detected in samples of both classic KS and AIDS-KS [5,6] (Figure 1E) and is considered to play an important role in the pathogenesis of AIDS-KS [5], we explored whether BP1 is expressed in AIDS-KS tumors. BP1 expression was detected in keratinocyes, spindle cells and in mononuclear cells infiltrating KS tumors. The BP1 protein was visualized by immunohistochemistry (Figure 1) and the mRNA by in situ hybridization (Figure 2). The expression of BP1 was not limited to KS lesions in the skin but was also detected in KS lesions in the intestine (data not shown). BP1 expression was higher in focal KS tumor areas and infiltrating cells, when compared to normal skin and keratinocytes where BP1 expression is detectable in the absence of tumors [15,20] (data not shown). The expression of BP1 in KS spindle cells, as well as infiltrating mononuclear cells located in close proximity to tumor vessels, strongly suggests that local production and release of BP1 functions to enhance the angiogenic activity of FGF-2 in these structures.

### HIV-Tat competes with FGF-2 for binding to BP1

**Figure 3:**
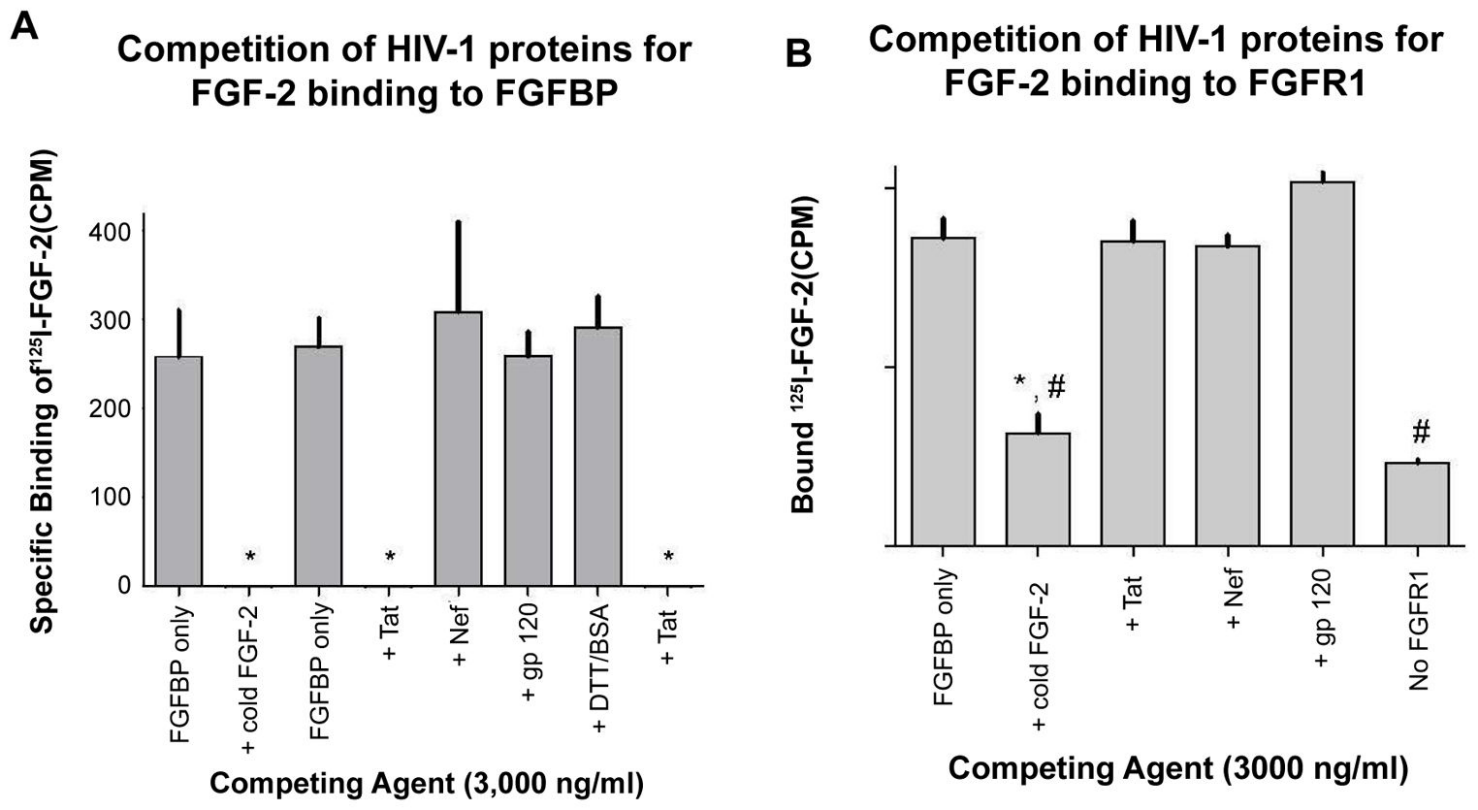
Effect of HIV-Tat protein on FGF-2 binding to BP1 (A) or FGF receptor (B) in cell-free binding assays with recombinant proteins **A**. Immobilized recombinant human BP1 protein was incubated with [125]-I-FGF-2 in the absence (control) or presence of different competitors: FGF-2, HIV-Tat, Nef and gp120. Data points represent specific binding of [125]-I-FGF-2 after subtraction of binding to wells coated with blocking solution only. Competition for [125]-I-FGF-2 binding was only observed with cold FGF-2 or HIV Tat protein as competitors. *, p<0.05 relative to “FGFBP only”. **B**. An FGF receptor-IgG fusion protein (500 ng/ml; FGFR1) and [125]-I-FGF-2 (20 ng/ml) were incubated with protein A/G agarose beads, in the absence (control) or presence of cold FGF-2, Tat, Nef, or gp120. One sample set with the receptor protein and radiolabeled FGF-2 was also run without the FGFR-IgG fusion protein (“No FGFR1”). Washed beads were collected and bound radioactivity was detected and is expressed relative to control. Bars are means of triplicates + SEM from one representative experiment. This experiment was repeated twice. There was no significant difference in receptor binding of [125]-I-FGF-2 in control samples versus those containing Tat, Nef, or gp 120. In the absence of FGF receptor protein or with added cold FGF-2 significantly less ligand binding was observed. *, p<0.05 relative to “FGFR1 only”; #, no significant difference between “+Cold FGF-2” and “No FGFR1”.

HIV-Tat protein, although initially described as a nuclear transcriptional regulatory protein, has also been shown to function as an angiogenic heparin-binding growth factor [12,13]. Moreover, Tat and FGF-2 synergize to induce an aggressive and highly angiogenic phenotype of AIDS-Kaposi’s sarcoma [5]. Therefore, we investigated its potential role in affecting the interactions between FGF-2 and BP1 *in vitro*. Two other HIV-proteins, Nef and gp120 were also tested to determine the specificity of the Tat effects in this process. As shown in Figure 3A, the HIV-Tat protein inhibited the binding of radiolabeled FGF-2 to the immobilized BP1 protein to the same extent as unlabeled (“cold”) FGF-2. In contrast, HIV-Nef and HIV-gp120 used at the same concentrations as HIV-Tat, had no effect on [125]I-FGF2 binding to immobilized BP1. DDT-treated BSA. BSA was used as a further negative control and showed no interference with the FGF-2 / BP1 interaction. We conclude from these findings that Tat binding to FGF-2 overlaps with FGF2 / BP1 interaction site(s) or generates a steric hinderance for FGF-2 binding.

### HIV-Tat protein does not interfere with FGF-2 binding to FGFR1

Since HIV-Tat protein acts as an angiogenic growth factor and can bind FGF-2, we carried out additional *in vitro* experiments to determine whether HIV-Tat may interfere with the binding of FGF-2 to FGFR1. In these experiments, [125]I-FGF2 was added to immobilized, recombinant FGFR1 protein in the absence or presence of Tat (>1,000-fold excess relative to the [125]I-FGF2 concentration). As shown in Figure 3B even an excess concentration of HIV-1 Tat protein does not inhibit FGF-2 binding to the FGFR1. In contrast, an excess concentration of cold FGF-2 blocked the binding of FGF-2 to the immobilized FGFR1 to background levels (Figure 3B, “No FGFR1”). Similar to Tat, the HIV-proteins Nef and gp120 also failed to inhibit FGF-2/FGFR1 interactions. From these data we conclude that Tat does not inhibit the interactions of FGF-2 with its high affinity receptor in a cell-free assay.

### Conditional BP1 transgene expression induces KS-like skin lesions during wound healing

**Figure 4:**
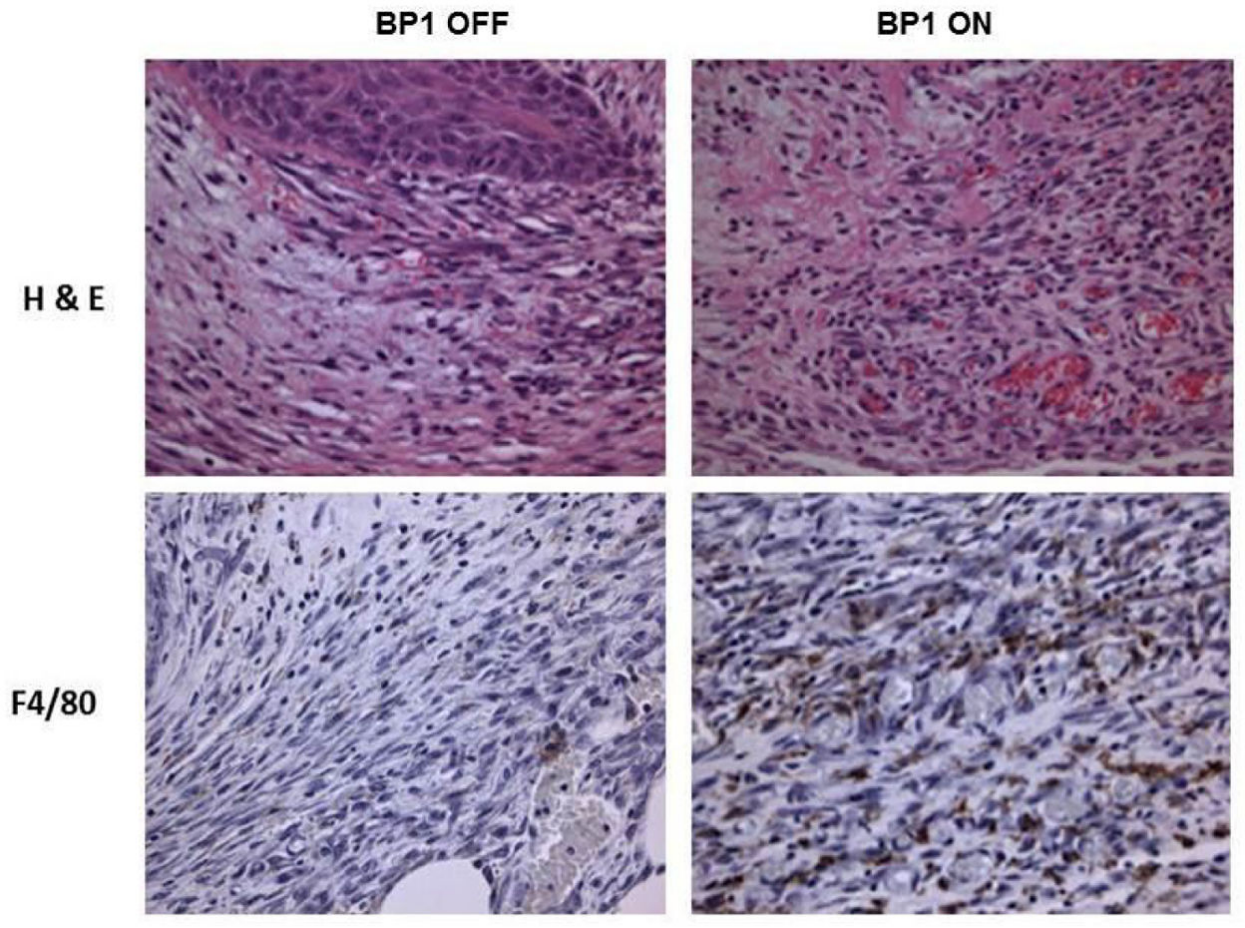
KS-like skin lesion in BP1 expressing transgenic mice The upper panels show representative hematoxylin and eosin (H&E) stained sections of excisional skin wounds from transgenic mice with the BP1 transgene OFF or ON four days after wound healing. The lower panels show representative macrophage (F4/80) stained sections of excisional skin wounds from transgenic mice with BP1 OFF or ON four days after the initial skin injury. Original magnification 250X.

A previous study by Tassi et al. showed that BP1 induced angiogenic lesions in the skin of BP1 ON conditional transgenic mice by 3-fold during wound healing [19]. This effect of BP1 on angiogenesis was strictly dependent on FGF signaling because systemic treatment of animals with an FGFR inhibitor (PD173074) prevented the induction of angiogenesis [19]. When we re-examined the histological samples from this study series, we found that many of the skin lesions show proliferating spindle cells, dilated capillaries, interstitial edema, and infiltrating inflammatory cells that resemble KS lesions (Figure 4).

## Discussion

In this study, we have described a potential novel mechanism by which the release and activity of FGF-2 can be modulated in AIDS-KS lesions. More specifically, we have found that a secreted FGF-binding protein (FGFBP1 or BP1) is produced by KS spindle cells, and by mononuclear cells infiltrating these vascular tumors. Since BP1 facilitates the release of FGF-2 from the extracellular matrix [15,16,20], and can modulate the binding interactions of FGF-2 with HSPG, these findings suggest that BP1 could potentially regulate the release and the paracrine activity of FGF-2 in KS lesions [21].

In general, the biological activity of FGF-2 is quenched by its tight binding to HSPG located in the extracellular matrix [22,23]. Thus, to become active, FGF-2 needs to be released and solubilized to reach its target cells in a paracrine activity mode [9]. One established mechanism that can solubilize FGF-2 from this storage site is the digestion of the glycosaminoglycan portion of the cell attachment molecule by heparanases [24–27]. Indeed, peripheral blood mononuclear cells (PBMC’s) can release heparanase activities that can in turn release active FGF-2 from the local storage in the extracellular matrix [25,28]. Furthermore, proteases that can digest the protein backbone of the HSPG could also release FGFs from the immobilized state and thus contribute to their activation [26,27]. For example, normal endothelial cells do not release FGF-2, do not proliferate in response to exogenous Tat, and do not induce the development of KS like lesions when injected into nude mice [28–30]. However, when normal endothelial cells are exposed to conditioned media from activated T cells, they acquire a spindle cell morphology, produce and release FGF-2 [29–31] and become responsive to HIV-Tat. Thus it is possible that proteolytic enzymes (i.e, plasmin, thrombin) [12,13] or chemokines (i.e., interferon γ) [29–31] produced by activated T cells could facilitate the release of FGF-2 from normal endothelial cells or other target cells. However, the extent of the role of heparanases, cytokines, and/or proteases for the release of FGF-2 in the context of KS, is not clearly defined at the present time.

Our data suggest an alternative mode of delivering active FGF-2 from the ECM storage site to its receptor could be via non-covalent binding to a secreted carrier protein. Such a secreted binding protein for FGFs was described by Sato and co-workers in 1991 [14]. This protein was purified from supernatants of A431 cells by virtue of its ability to bind with high affinity to FGF-2 using ligand affinity chromatography with immobilized FGF-2 as the crucial step [14]. BP1 contains a hydrophobic signal sequence and binds to FGF-1 and FGF-2 in a non-covalent, reversible manner [14], and releases immobilized FGF-2 from storage in the extracellular matrix of cultured cells that express both BP1 and FGF-2. This mobilized FGF-2 is biologically active [15,16]. Sato and coworkers also demonstrated that pre-incubation of FGF-2 with purified BP1 protein protects the mitogenic activity of FGF-2 from inactivation [14]. These characteristics make BP1 an excellent candidate carrier molecule for FGF-2. BP1 can also support tumor growth and angiogenesis of FGF-2 expressing non-tumorigenic SW-13 cells [15]. Expression of this binding protein in cell lines which express FGF-2 leads to a tumorigenic and angiogenic phenotype [15,16]. Moreover, BP1 transfected cells release the protein into their media together with FGF-2 in a non-covalently bound form. This released FGF-2 becomes activated biologically, and the growth phenotype of cells expressing BP1 can be blocked by a specific antibody for FGF2 [15,16]. Taken together, these studies support the notion that the secretion of BP1 may be an important activating step for the locally stored FGF-2 in KS lesions.

Previous studies have shown that HIV-Tat synergizes with FGF-2 to induce the proliferation of endothelial and KS spindle cells. More recently we found that the heparin binding motif of HIV-Tat plays a crucial role facilitating its recruitment to lipid rafts and induction of FGF-2 signaling [32]. Here, we report that HIV-Tat competes with FGF-2 for binding to BP1, but does not disrupt the binding of FGF-2 to it’s high affinity receptor FGFR1 (Figure 3). Thus, an equilibrium must exists in the extracellular matrix of KS tumors, keeping FGF-2 bound to immobilized HSGPs. In this manner, HSPGs act as a depot that keep free FGF-2 at a sufficiently low concentration, preventing target cell receptor activation. Our findings show that BP1 can be produced by multiple KS cells, including infiltrating mononuclear cells located in close proximity to the tumor vessels. Thus, since BP1 is a soluble carrier protein that can bind FGF-2 molecules available in solution, it might also release FGF-2 molecules from HSPGs located on the cell surface of tumor vascular endothelial cells. Moreover, BP1 reduces the binding affinity of FGF-2 for HSPGs [14–16] and by this BP1 can amplify the angiogenic activity of FGF-2 in the KS tumor environment. Finally, it is worth noting that our results clearly show that the expression of BP1 is not limited to AIDS-KS lesions. BP1 was also detected in similar locations in the skin of HIV-negative KS tumors, as well as in injured tissues from HIV-infected patients without KS (e.g. kidney). Furthermore, in a previous study we showed that BP1 staining was significantly up-regulated in pre-malignant human skin lesions and during wound healing when compared to normal skin [33]. Moreover, both FGF-2 and BP1 are produced by infiltrating mononuclear cells [7], which are not detected in normal skin, providing an additional source of BP1 and FGF-2 release during inflammation. Thus, BP1 may play a more general role modulating the angiogenic activity of FGF-2 in different tissues of HIV-infected patients undergoing inflammatory lesions, including the kidney [17].

The protein coding sequence of the 234 amino acid FGFBP1 is contained in a single exon and the gene is located on chromosome 4 in human and the respective syntenic loci in other vertebrates [34]. We identified the C-terminal 44 amino acids in FGFBP1 as the FGF binding domain [35]. Other portions of the protein interact with heparin. Interestingly, cysteins positions in the protein are conserved across species and can form disulfide bridges that will stabilize the protein when bound to an FGF [35]. Amino acid sequence and protein structure comparisons of secreted FGFBP1 with the well-defined FGF binding domain in the transmembrane FGF receptors did not reveal any common features of the domains [9,35]. This suggests that the respective FGF binding domains in FGFBP1 and in FGF receptors evolved independently and will bind to distinct surfaces on FGFs. Furthermore, in the presence of FGFBP1 an enhanced receptor signaling activity of FGFs was observed supporting the notion that binding of FGFs to their receptor and to FGFBP1 do not overlap or generate a steric hindrance of ligand access to the receptor [9,15,19].

In summary, we have shown that a FGF binding protein (FGFBP1) which is known to positively modulate the angiogenic and growth promoting activity of FGF’s in tumor cells [15,16], is expressed in keratinocytes, infiltrating mononuclear cells, and KS spindle cells. In addition, we found that the HIV-Tat can modulate the binding interactions of BP1 and FGF-2. Finally, we found that activation of BP1 during the process of wound healing in the skin, can induce angiogenic lesions that resemble KS. Taken together, when these findings are interpreted in the context of previous studies, they provide a novel mechanism by which the activity of FGF-2 and HIV-Tat can be modulated in AIDS-KS tumors.
